# Bovine colostrum–derived extracellular vesicles modulate gut microbiota and alleviate atopic dermatitis via the gut–skin axis

**DOI:** 10.1007/s13346-025-01875-z

**Published:** 2025-05-15

**Authors:** Daye Mun, Sangdon Ryu, Hyejin Choi, Min-Jin Kwak, Sangnam Oh, Younghoon Kim

**Affiliations:** 1 Department of Agricultural Biotechnology and Research Institute of Agriculture and Life Science, Seoul National University, Seoul, 08826 Korea; 2 Honam National Institute of Biological Resources, Mokpo, 58762 Korea; 3 Emerging Pathogens Institute, Department of Animal Sciences, University of Florida, Gainesville, FL 32611 USA; 4 Department of Functional Food and Biotechnology, Jeonju University, Jeonju, 55069 Korea

**Keywords:** Bovine colostrum–derived extracellular vesicles, Atopic dermatitis, Gut–skin axis, Gut microbiota, Gut metabolomics, Immune response

## Abstract

Atopic dermatitis (AD) is a chronic inflammatory skin condition characterized by immune dysregulation and a disrupted gut–skin axis. Emerging evidence suggests that the gut microbiota and their metabolites play a critical role in pathogenesis and potential treatment of AD. However, therapeutic strategies targeting the gut microbiota that aim to alleviate AD remain underexplored. Therefore, this study investigated the potential of bovine colostrum–derived extracellular vesicles (BCEVs) to ameliorate AD symptoms by modulating the gut microbiota and intestinal metabolites. AD was induced in mice using 2,4-dinitrochlorobenzene, followed by the oral administration of BCEVs. Skin lesions were assessed histologically to evaluate disease severity. Allergic and immune responses were measured by analyzing serum immunoglobulin E (IgE) levels and cytokine profiles, including interleukin-4 (IL-4) and tumor necrosis factor-alpha (TNF-α). Gut microbiota composition was determined using 16 S rRNA gene sequencing, and the metabolomic profiling of intestinal samples was performed using gas chromatography–mass spectrometry to identify metabolites. BCEV treatment significantly alleviated skin lesions and reduced the serum IgE levels and the imbalance in IL-4 and TNF-α levels associated with AD induction. Gut microbiota analysis revealed that BCEVs restored microbial dysbiosis and improved the abundance of beneficial bacteria, and metabolomic analysis demonstrated elevated levels of lactic acid and other metabolites. These findings suggest that BCEVs alleviate AD symptoms by rebalancing the gut microbiota and intestinal metabolomes. This study emphasizes the importance of targeting the gut–skin axis as a novel strategy for AD treatment and provides evidence for the therapeutic potential of BCEVs in skin-related immune disorders.

**Graphical abstract:**

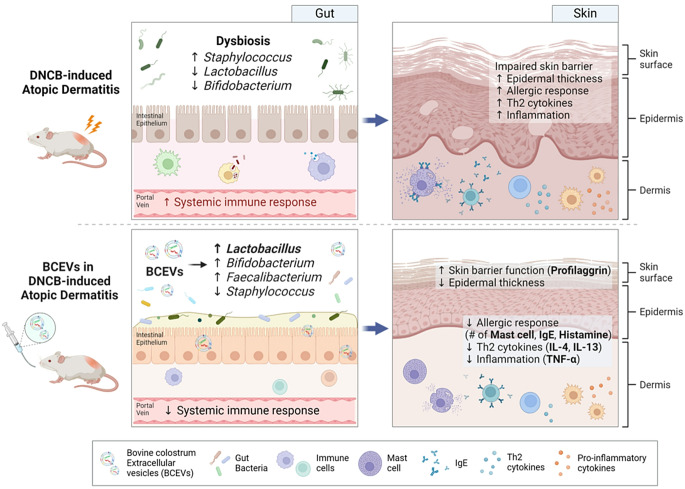

## Introduction

Atopic dermatitis (AD) is a chronic inflammatory skin disease characterized by dry, itchy, and inflamed skin [1]. Often beginning in early childhood, this condition tends to follow a relapsing–remitting course, profoundly impacting the quality of life of those affected because of persistent discomfort [2]. The pathophysiology of AD is complex, involving genetic, environmental, and immunological factors [3]. The key feature of the condition is impaired skin barrier function, which accelerates moisture loss and makes the skin more vulnerable to irritants and allergens [4]. This compromised barrier also predisposes the skin to bacterial, viral, and fungal infections, which can exacerbate the condition [1]. Histologically, AD is characterized by epidermal hyperplasia, hyperkeratosis, immune cell infiltration, and dermal edema, which reflect the chronic inflammatory state of the skin [5]. The immunological pathogenesis of AD includes increased immunoglobulin E (IgE) levels in the blood, increased eosinophil count, and increased Th2 cytokine levels, including those of interleukin-4 (IL-4), IL-5, and IL-13 [6].

The concept of the gut–skin axis, which suggests that the health of the gastrointestinal tract can influence systemic inflammation and, consequently, skin health, offers an interesting direction for understanding and managing AD [7]. The axis is based on the principle that the gut microbiome plays an important role in maintaining gut and systemic health [8]. Gut dysbiosis is associated with increased intestinal permeability that allows toxins and pathogens to escape into the bloodstream [9] and potentially cause systemic inflammation that can worsen skin conditions such as AD [10]. Therefore, in recent years, there has been increasing research into targeting gut microbiota to alleviate AD.

Bovine colostrum–derived extracellular vesicles (BCEVs) are important components of cell-to-cell communication and immune regulation. These vesicles are bilipid membrane structures filled with proteins, lipids, and microRNAs, which are involved in regulating gene expression and altering cellular function throughout the body [11]. These properties have prompted growing interest in the therapeutic potential of BCEVs for various diseases. In particular, several studies have demonstrated that milk- and colostrum-derived EVs can modulate the gut microbiota by delivering bioactive molecules such as miRNAs, proteins, and lipids [12–15]. Dietary supplementation with bovine milk EVs has been shown to alter gut microbial composition, increase the abundance of beneficial bacteria, and regulate microbial metabolites including short-chain fatty acids [12–14]. Moreover, BCEVs have been reported to improve gut barrier function, alleviate gut inflammation, and modulate immune responses in colitis models [14–18]. Given the emerging concept of the gut–skin axis, modulation of gut microbiota by BCEVs is expected not only to improve gut health but also to exert beneficial effects on inflammatory skin diseases such as AD. However, to the best of our knowledge, no studies have investigated the potential of milk- or colostrum-derived EVs in mitigating AD symptoms through gut microbiota modulation.

Unlike whole colostrum or colostrum-based supplements, BCEVs offer several distinct advantages, including enhanced stability during storage, low immunogenicity, and the potential for targeted delivery of bioactive cargo [19, 20]. Their encapsulated nature protects functional molecules such as miRNAs and proteins from degradation and enables more precise modulation of host responses and gut microbiota [21–23]. Therefore, this proof-of-concept study aimed to investigate whether BCEVs could alleviate AD symptoms through gut microbiota modulation, highlighting their potential as bioactive components for preventing or alleviating inflammatory diseases.

## Experimental section

### EV isolation

EVs were isolated from colostrum samples following a modified protocol based on the method previously described [24]. Briefly, colostrum fat was separated by centrifugation at 1,200 × g for 10 min. The supernatant was subsequently centrifuged at 16,000 × g for 1 h to remove cellular debris. The resulting supernatant was subjected to ultracentrifugation at 50,000 × g for 1 h to obtain the whey fraction. Large particles were removed from the whey fraction by ultracentrifugation at 100,000 × g. EVs were pelleted by further ultracentrifugation of the supernatant at 135,000 × g for 90 min. To ensure purity, the EV pellet was washed with phosphate-buffered saline (PBS), and centrifugation steps were repeated. Finally, the purified EVs were resuspended in PBS and stored at − 80 °C until further analysis. The EVs used in this study were prepared using the same protocol and source material as in our previous study, where their characteristics were thoroughly evaluated according to MISEV2023 guidelines, including size, morphology, and marker protein expression [25].

### Animals and treatment

All animal experiments were performed after review and approval by the Institutional Animal Care and Use Committee of Seoul National University (SNU-221101-2-2). Seven-week-old male mice (BALB/c) were purchased from OrientBio (Seongnam, Korea) and housed under constant conditions (12-h light/dark cycle) at 55% ± 5% humidity and 22 °C ± 1 °C. Water and food were provided *ad libitum* to the mice during the experimental period. Mice were allowed to adapt to the experimental environment for 7 days prior to treatment. A total of 15 mice were used in the experiments and randomly assigned to three groups (*n* = 5 per group): CON (PBS-treated control), DNCB (2,4-dinitrochlorobenzene-induced atopic dermatitis control + PBS), and BCEVs (DNCB + BCEVs 1 × 10^11^ particles/mouse). The back skin was shaved 24 h prior to DNCB application to ensure effective transdermal delivery while minimizing barrier damage. For AD induction, 1% DNCB was applied to the back and ears on days 0 and 3, the sensitization period. For the next 2 weeks, 0.5% DNCB was applied every other day as a challenge period [26, 27]. DNCB was dissolved in a 3:1 mixture of acetone and olive oil (Sigma, St. Louis, MO, USA). BCEVs were administered orally daily for 2 weeks after sensitization. To compare the effects of oral and topical administration of BCEVs, an additional experiment was conducted using the same number of mice (*n* = 5 per group) and treatment conditions as the oral administration group. Following the induction of AD using the same DNCB sensitization and challenge protocol described above, BCEVs (1 × 10¹¹ particles/mouse) or PBS were applied topically to the dorsal skin once daily for 2 weeks. Ear thickness measurements and dermatitis scores were assessed every other day, and criteria were based on previous publications [28]. Ear thickness was measured using a digital caliper, and dermatitis severity was evaluated by scoring four parameters—erythema/hemorrhage, scarring/dryness, edema, and excoriation—each on a scale from 0 (none) to 3 (severe), with a maximum total score of 12 per mouse.

### Tissue staining

After the mice were sacrificed, the back skin was dissected, fixed in 4% paraformaldehyde (PFA), and processed for paraffin embedding. Sections of 4 μm thickness were cut from the paraffin blocks and subjected to hematoxylin and eosin (H&E) and toluidine blue staining. Histopathological features were examined under a light microscope, and the epidermal thickness and number of mast cells were measured using ImageJ software (NIH, Bethesda, MD, USA) by selecting five random regions per section. All measurements were performed in a blinded manner, and the average values were used for statistical analysis.

### Serum ELISA

Blood was collected from mice before sacrifice, and serum was isolated. The amounts of serum IgE and histamine were measured using a mouse IgE ELISA kit and a histamine ELISA kit (Abcam, UK).

### RNA isolation and qRT‑PCR analysis

Total RNA was isolated from 30 mg of skin tissue using TRIzol reagent (Invitrogen, USA) and an RNeasy Plus kit (Qiagen, Hilden, Germany) according to the recommended protocol. RNA concentration and purity were assessed by measuring absorbance using a Spectramax ABS Plus spectrophotometer (Molecular Devices, San Jose, CA, USA). cDNA was synthesized using an iScript cDNA synthesis kit (Bio-Rad, CA, USA). qRT-PCR analysis was performed using SsoAdvanced Universal SYBR Green Supermix (Bio-Rad, CA, USA) through the CFX96™ system (Bio-Rad, CA, USA). The primer sequences applied for qRT-PCR are shown in Table 1.

**Table 1:** Primers used in this study for qRT-PCR

Primer	Sequence (5′–3′)
Gapdh	Forward: CATCACTGCCACCCAGAAGACTG Reverse: ATGCCAGTGAGCTTCCCGTTCAG
Tnf	Forward: GGTGCCTATGTCTCAGCCTCTT Reverse: GCCATAGAACTGATGAGAGGGAG
Il4	Forward: ACGGGAGAAGGGACGCCAT Reverse: GAAGCCGTACAGACGAGCTCA
Il13	Forward: CCTGGCTCTTGCTTGCCTT Reverse: GGTCTTGTGTGATGTTGCTCA
Flg	Forward: GAATCCATATTTACAGCAAAGCACCTTG Reverse: GGTATGTCCAATGTGATTGCACGATTG
Fcer1g	Forward: TCTCTTCTTCCAGCCTCCTTTGCT Reverse: TTGAGTCAGGTCTCTGGCAGCTTT

### Metagenomic analysis

DNA from cecal contents was extracted using a PowerFecal DNA isolation kit (Qiagen, Hilden, Germany). The V3–V4 region of the 16 S rRNA gene amplified through PCR was sequenced using Nextseq (2 × 300; Illumina Inc., San Diego, CA, USA) at Sanigen, Korea, according to the manufacturer’s instructions. Demultiplexing and QC were performed using raw data. Adaptor trimming, quality trimming, and read error correction were performed, and chimeric sequences were removed. Amplicon sequence variation was obtained using the split amplicon denoising algorithm 2 (DADA2) of the QIIME 2 plugin. For taxonomic analysis, classification was performed based on SILVA 138.1, alignment was performed after removing the mitochondrial and chloroplast sequences, and alpha and beta diversities were analyzed. Differences in genera between groups were determined using Welch’s t-test.

### Metabolomic analysis

Metabolome extraction, derivatization, and GC–MS analysis of each cecal content were performed as previously described with minor modifications [29]. Cecal samples were diluted in methanol to a final concentration of 100 mg/mL. After vortexing for 5 min, the samples were centrifuged at 15,000 × g for 5 min. The resulting supernatants were filtered, and 200 µL aliquots were concentrated using a vacuum concentrator. Enriched metabolites were subjected to derivatization using methoxyamine and N, O-bis (trimethylsilyl) trifluoroacetamide (BSTFA; Sigma, St. Louis, USA). To normalize quantification, fluoranthene was added to the extract as an internal standard. GC–MS analysis was performed using a Thermo Trace 1310 GC (Waltham, MA, USA) coupled to a Thermo ISQ LT single quadrupole mass spectrometer (Waltham, MA, USA). Separation was performed using a DB-5MS column (Agilent, Santa Clara, CA, USA) with dimensions of 60 m in length, 0.2 mm inner diameter, and a film thickness of 0.25 μm. Sample was injected at 300 °C, using a 1:5 split ratio and helium (7.5 mL/min) as the split carrier gas. Metabolite separation was carried out using a constant helium flow of 1.5 mL/min, with the following oven temperature program: an initial hold at 50 °C for 2 min, ramping to 180 °C at 5 °C/min (held for 8 min), then increasing to 210 °C at 2.5 °C/min, and finally reaching 325 °C at 5 °C/min with a final hold of 10 min. Mass spectral data were collected across a scan range of m/z 35 to 650, with an acquisition rate of 5 spectra per sec. Electron impact ionization was employed, and the temperature for the ion source was set to 270 °C. The spectra were processed using the AMDIS (Automated Mass Spectral Deconvolution and Identification System) software, which performed automatic peak detection and deconvolution. The detected metabolites were confirmed by matching mass spectra using the NIST Mass Spectrum Search Program (version 2.0, Gaithersburg, MD, USA). Further statistical analysis was performed using MetaboAnalyst 6.0.

### Statistical analysis

Statistical analyses were conducted using GraphPad Prism 10.0 software (GraphPad Software, San Diego, CA, USA). Differences between groups were assessed using Student’s t-test. Multiple group comparisons were performed using one-way ANOVA, followed by Tukey’s post hoc test. Welch’s t-test was performed using Statistical Analysis of Taxonomic and Functional Profiles (STAMP). Results were considered statistically significant at a p-value of < 0.05. All data were presented as mean ± standard deviation.

## Results

### BCEVs alleviated AD symptoms in DNCB-induced mice

**Fig. 1:**
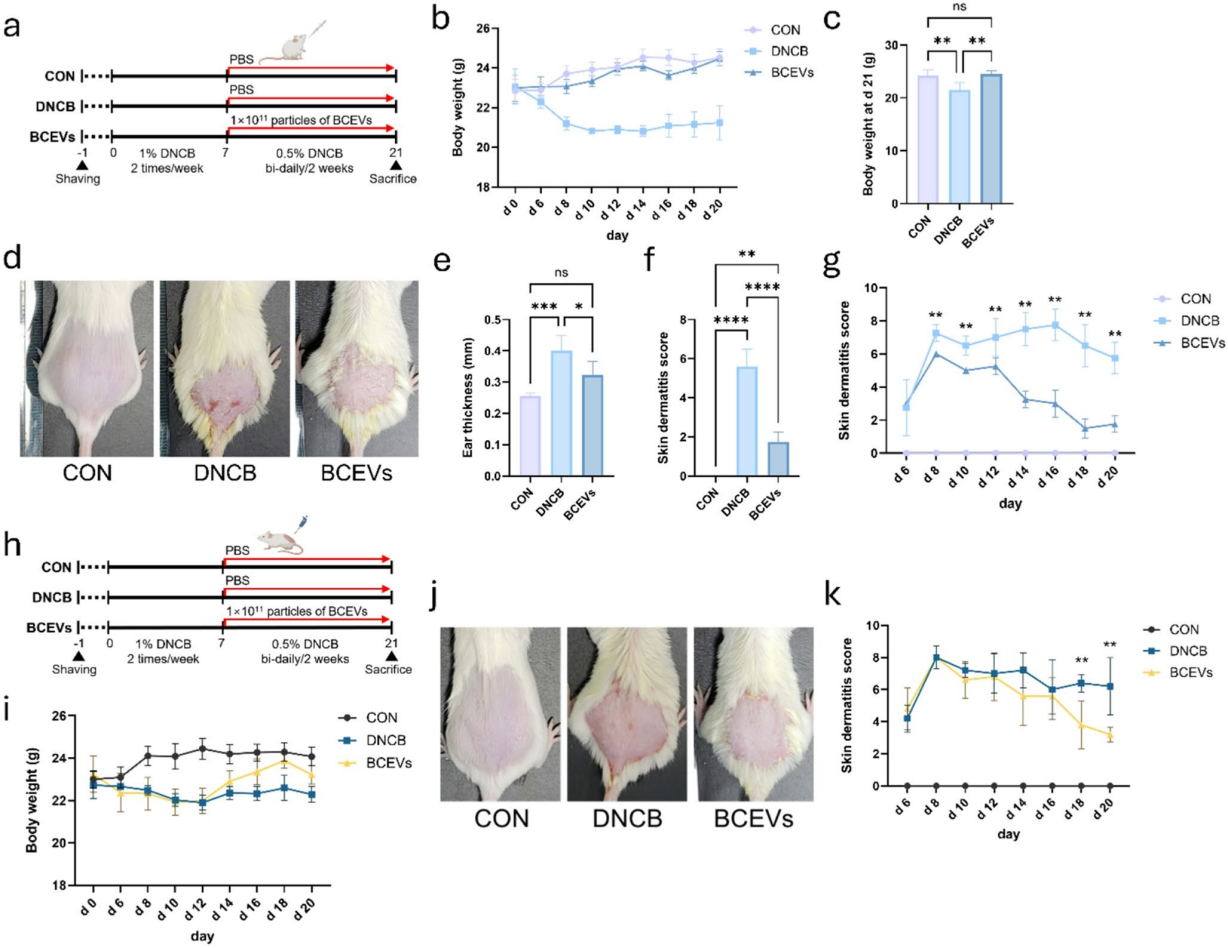
BCEVs modulate AD-like pathophysiology via oral or topical administration in mice. (**a**) Schematic of BCEV treatment by oral gavage (1 × 10^11^ particles/mouse). (**b**)–(**c**) Body weight changes in mice during BCEV treatment for 3 weeks (b) and body weight at d 21 (**c**). (**d**) Representative image of back skin taken on day 21. (**e**) Ear thickness. (**f**) Skin dermatitis score. (**g**) Skin dermatitis score during the experiment of oral gavage. (**h**) Schematic of EV treatment by topical exposure (1 × 10^11^ particles/mouse). (**i**) Body weight changes in mice during BCEV treatment for 3 weeks. (**j**) Representative image of back skin taken on day 21. (**k**) Skin dermatitis score during the experiment of topical treatment. All data represent the mean ± standard deviations (*n* = 5). One-way ANOVA and Tukey’s test analyzed differences among multiple groups. Asterisks represent statistical significance at *p* < 0.05 (*), *p* < 0.01 (**), *p* < 0.001 (***), and *p* < 0.0001 (****), while *ns* indicates not significant. AD, atopic dermatitis; CON, saline control; DNCB, AD control; BCEVs, AD induction with BCEVs

To determine the potential anti-inflammatory effects of BCEVs, they were orally administered to a DNCB-induced AD mouse model. The experimental design is shown in Fig. 1a. During the experimental period, the DNCB group showed a significant decrease in body weight compared with that of the other groups (Fig. 1b, c). When AD-like skin inflammation was visually assessed, DNCB treatment significantly increased AD-like skin inflammation and the ear thickness and skin dermatitis score were significantly increased (Fig. 1d–f). By contrast, in the BCEV-treated group, body weight was restored and both the ear thickness and skin dermatitis score were significantly reduced. To determine whether applying BCEVs directly to the skin would achieve the same effect, BCEVs were applied directly to the back skin of DNCB-treated mice to assess whether the skin lesions improved (Fig. 1h). Results showed that on day 21, the last day of the experiment, the mice on which BCEVs were topically applied appeared to recover, similar to the oral group; however, the recovery period was later than that of the oral treatment group (Fig. 1g and i–k). These results suggest that the oral administration of BCEVs could effectively ameliorate DNCB-induced AD rather than direct wound exposure.

### BCEVs alleviate skin lesions and allergic response in DNCB-induced AD-like mouse model

**Fig. 2:**
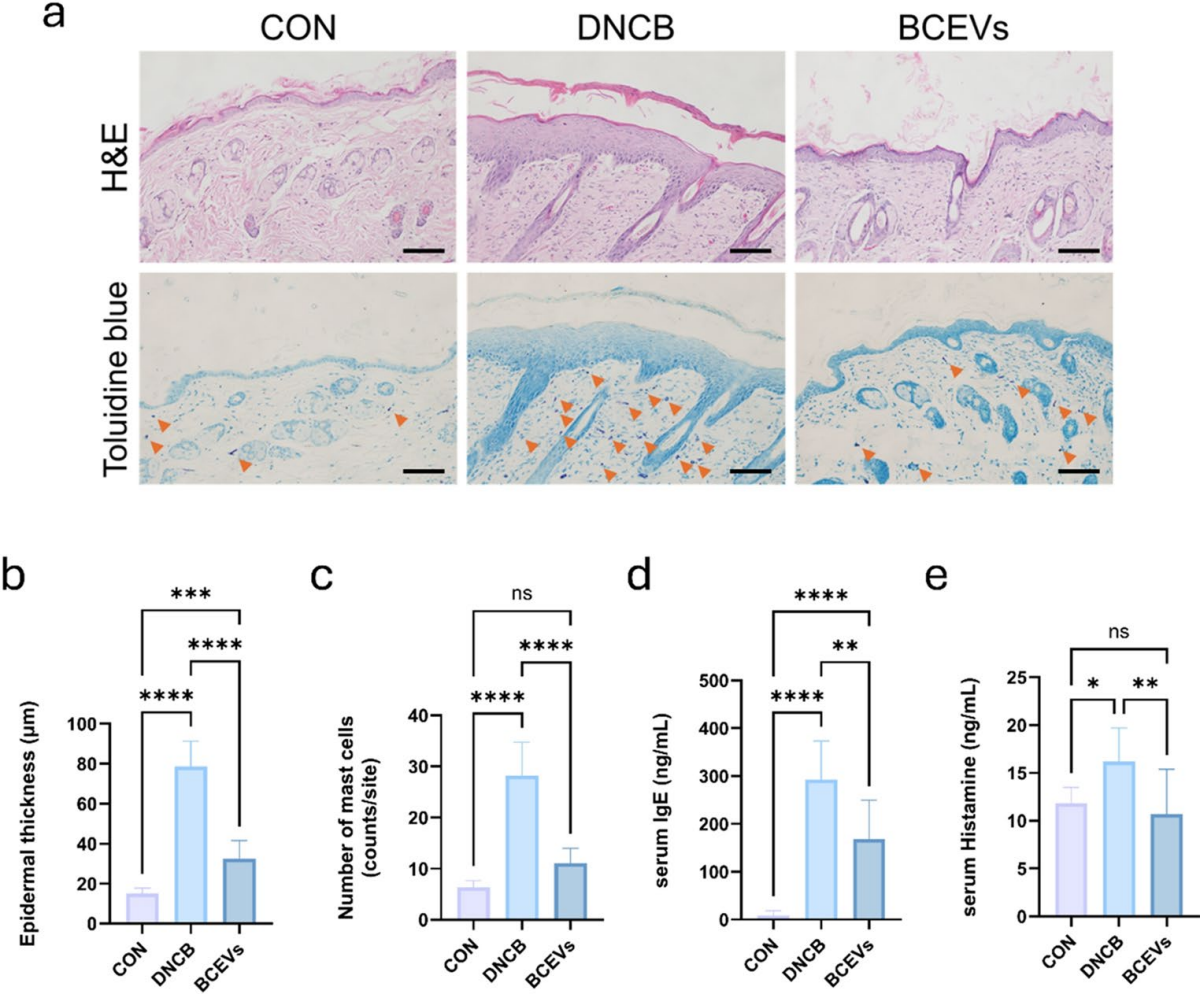
Skin lesions and allergic responses in AD-like mouse model are improved by BCEVs. (**a**) Representative histological sections of the skin. Scale: 100 μm. (**b**) Epidermal thickness. (**c**) Number of mast cells. (**d**)–(**e**) Serum levels of IgE (**d**) and histamine (**e**), the biomarkers of the allergic response. The orange arrow indicates mast cells. All data represent the mean ± standard deviations (*n* = 5). One-way ANOVA and Tukey’s test analyzed differences among multiple groups. Asterisks represent statistical significance at *p* < 0.05 (*), *p* < 0.01 (**), and *p* < 0.0001 (****), while *ns* indicates not significant. AD, atopic dermatitis; CON, saline control; DNCB, AD control; BCEVs, AD inducing with BCEVs

To determine whether the oral administration of BCEVs affected skin thickness alteration and AD-like allergic response, skin tissue staining was performed. H&E staining revealed that epidermal thickness, which was significantly increased in the DNCB group, was significantly reduced by BCEVs (Fig. 2a, b). In addition, mast cell count was measured using toluidine blue staining, and BCEVs significantly reduced the number of AD-induced increase in mast cells (Fig. 2c). To determine whether the increased number of mast cells affected the release of cytokines and histamine, the amounts of IgE and histamine in the serum were quantified (Fig. 2d, e). The results showed that IgE and histamine were detected at higher levels in the DNCB group, which were reduced by the administration of BCEVs, consistent with the previous results.

### BCEVs modulate immune response in DNCB-induced AD-like mouse model

**Fig. 3:**
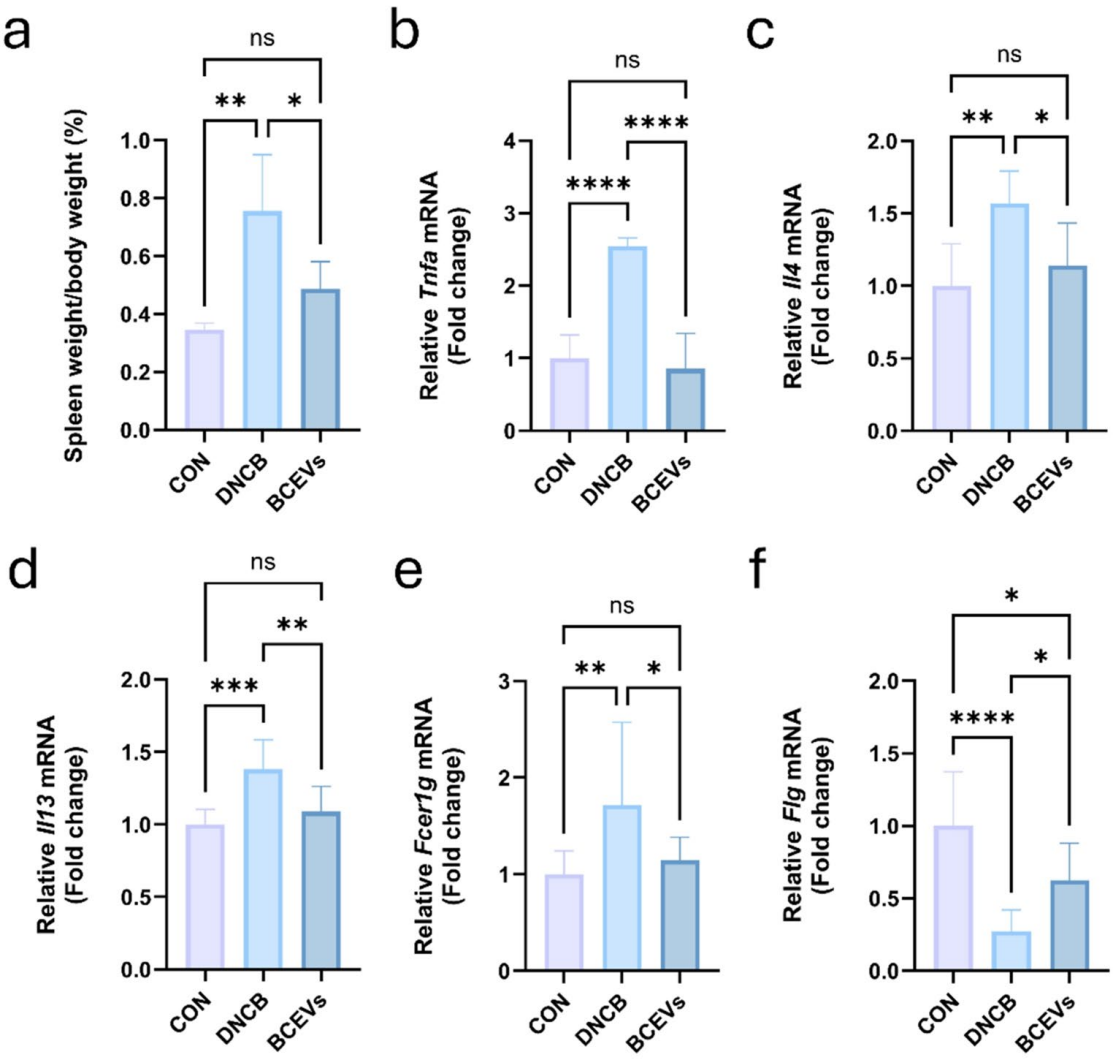
BCEVs modulate inflammation-related mRNA expression in AD-like mouse model. (**a**) Spleen weight. (**b**)–(**f**) Fold change in mRNA expression of *Tnfa* (**b**), *Il4* (**c**), *Il13* (d), *Flg* (**e**), and *Fcer1g* (**f**). All data represent the mean ± standard deviations (*n* = 5). One-way ANOVA and Tukey’s test analyzed differences among multiple groups. Asterisks represent statistical significance at *p* < 0.05 (*), *p* < 0.01 (**), *p* < 0.001 (***), and *p* < 0.0001 (****), while *ns* indicates not significant. AD, atopic dermatitis; CON, saline control; DNCB, AD control; BCEVs, AD inducing with BCEVs

To investigate how the oral administration of BCEVs affected the immune response, the expression of cytokines in skin tissues was measured by qPCR. Spleen weight, a marker of the systemic immune response, was significantly increased in the DNCB group (Fig. 3a), and *Tnfa*, *Il4*, *Il13*, and *Fcer1g* were significantly increased in the skin sample (Fig. 3b–e). By contrast, BCEV administration significantly decreased both spleen weight and *Tnfa*, *Il4*, *Il13*, and *Fcer1g*, indicating that BCEVs have the effect of alleviating inflammatory and allergic reactions. The expression of *Flg*, a gene encoding profilaggrin, which is essential in the skin barrier, was significantly decreased by AD induction, which was significantly increased by BCEV treatment (Fig. 3f).

### BCEVs improve gut dysbiosis in DNCB-induced AD-like mouse model

**Fig. 4:**
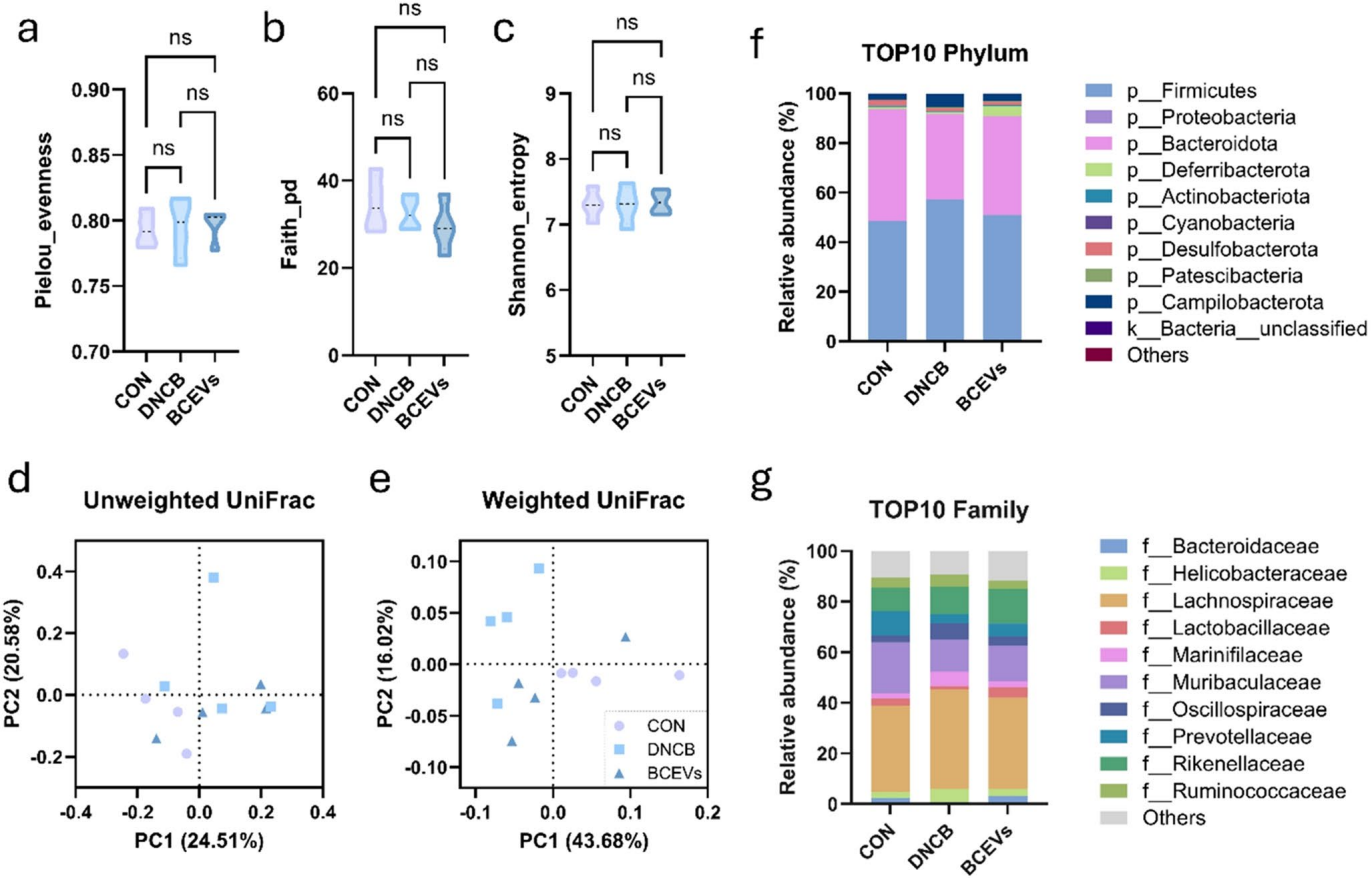
BCEVs restore gut microbiota imbalance in AD-like mouse model. (**a**)–(**c**) Alpha diversity analysis using the Pielou evenness index (**a**), Faith pd index (**b**), and Shannon entropy index (**c**). (**d**)–(**e**) Beta diversity analysis using unweighted UniFrac (**d**) and weighted UniFrac (**e**) indices. (**f**)–(**g**) Related abundance (%) of the top 10 phylum (**f**) and top 10 family (**g**) of the gut microbiota. All data represent the mean ± standard deviations (*n* = 4, randomly selected from 5). One-way ANOVA and Tukey’s test analyzed differences among multiple groups. *ns* indicates not significant. AD, atopic dermatitis; CON, saline control; DNCB, AD control; BCEVs, AD inducing with BCEVs

Metagenomic analysis was performed on cecal content to determine whether the AD-attenuating effects of BCEVs were associated with the gut microbiota. Alpha diversity analysis showed no significant differences among groups in all three factors (Fig. 4a–c). Beta diversity analysis revealed no clustering in the unweighted UniFrac (Fig. 4d), which considers only qualitative variables, whereas clustering was observed in the weighted UniFrac (Fig. 4e), where quantitative variables are assessed. This suggests that treatment with BCEVs did not affect the qualitative aspects of the gut microbiota but did affect the abundance of each bacteria.

**Fig. 5:**
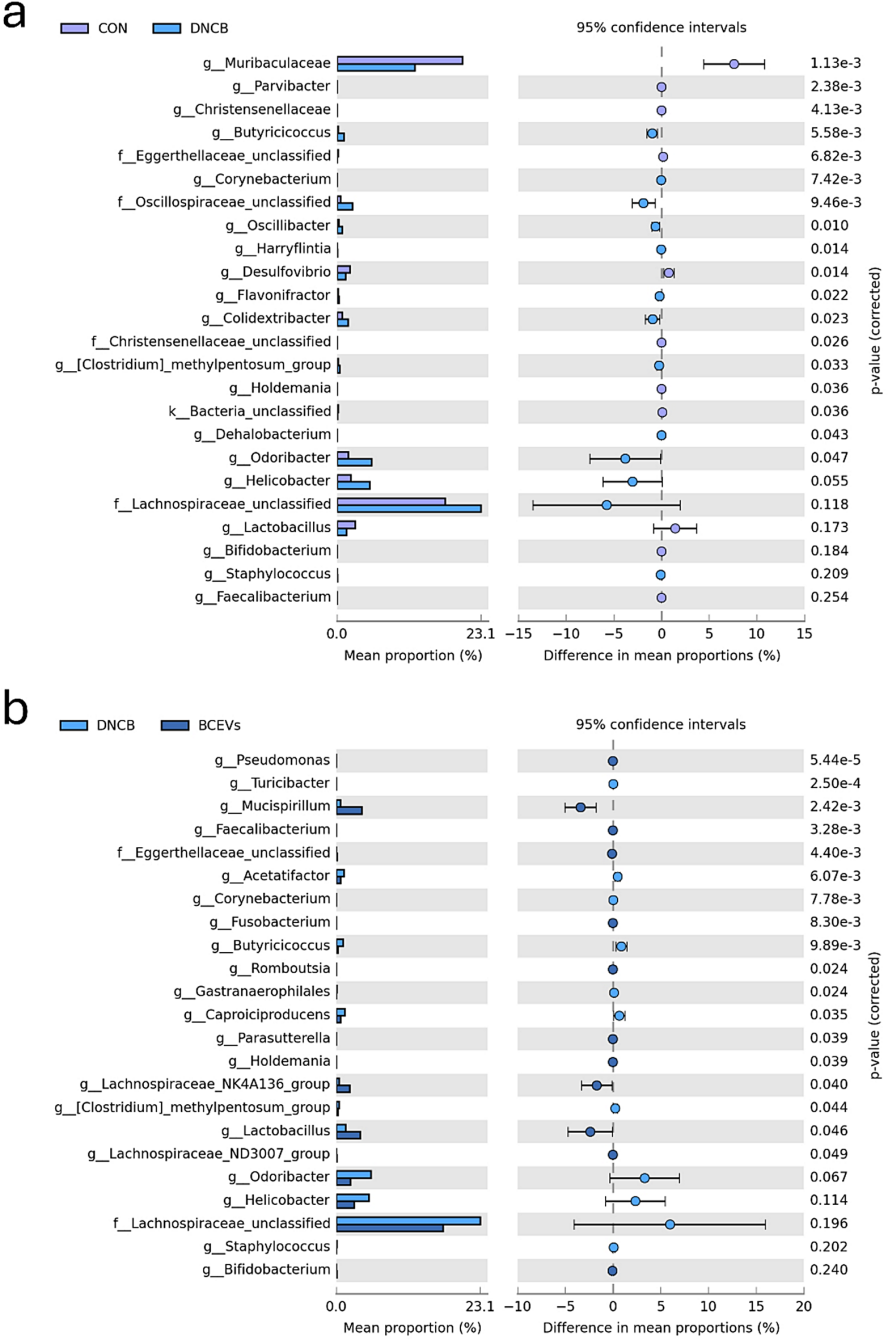
Comparison of significantly altered genera in the gut microbiota. (**a**)–(**b**) Differential abundance analysis between CON and DNCB group (**a**) and between DNCB and BCEVs group (**b**). Statistical significance was determined using Welch’s t-test. AD, atopic dermatitis; CON, saline control; DNCB, AD control; BCEVs, AD inducing with BCEVs

At the phylum level, Firmicutes showed a slight increase in the DNCB group, and Bacteroidota tended to decrease (Fig. 4f). This change in the ratio of Firmicutes to Bacteroidota appeared to be slightly ameliorated in the BCEV group. At the family level (Fig. 4g), Muribaculaceae, Prevotellaceae, and Lactobacillaceae decreased in the DNCB group, Lachnospiraceae, Oscillospiraceae, and Helicobacteraceae increased. In the BCEV group, Lachnospiraceae, Prevotellaceae, and Muribaculaceae decreased numerically, although not significantly, and Lactobacillaceae increased significantly. In addition, at the genus level, the abundance of *Oscillibacter*, *Corynebacterium*, *Odoribacter*, and [*Clostridium*]_*methylpentosum*_*group* were significantly increased in the DNCB group, and *Helicobacter*,* Staphylococcus*, and *Lachnospiraceae*_*unclassified* were numerically increased compared to the control group (Fig. 5a). Conversely, the abundance of *Lactobacillus*, *Faecalibacterium*, *Parasutterlla*, and *Mucispirillum* was significantly increased in the BCEV-treated group, and *Bifidobacterium* also showed a numerical but not significant increase (Fig. 5b). Furthermore, *Corynebacterium* and *[Clostridium]_methylpentosum_group* were significantly decreased in the BCEV group, while *Odoribacter*, *Helicobacter*, and *Staphylococcus* were numerically decreased compared to the DNCB group (Fig. 5b).

### BCEVs alter the gut metabolites in DNCB-induced AD-like mouse model

**Fig. 6:**
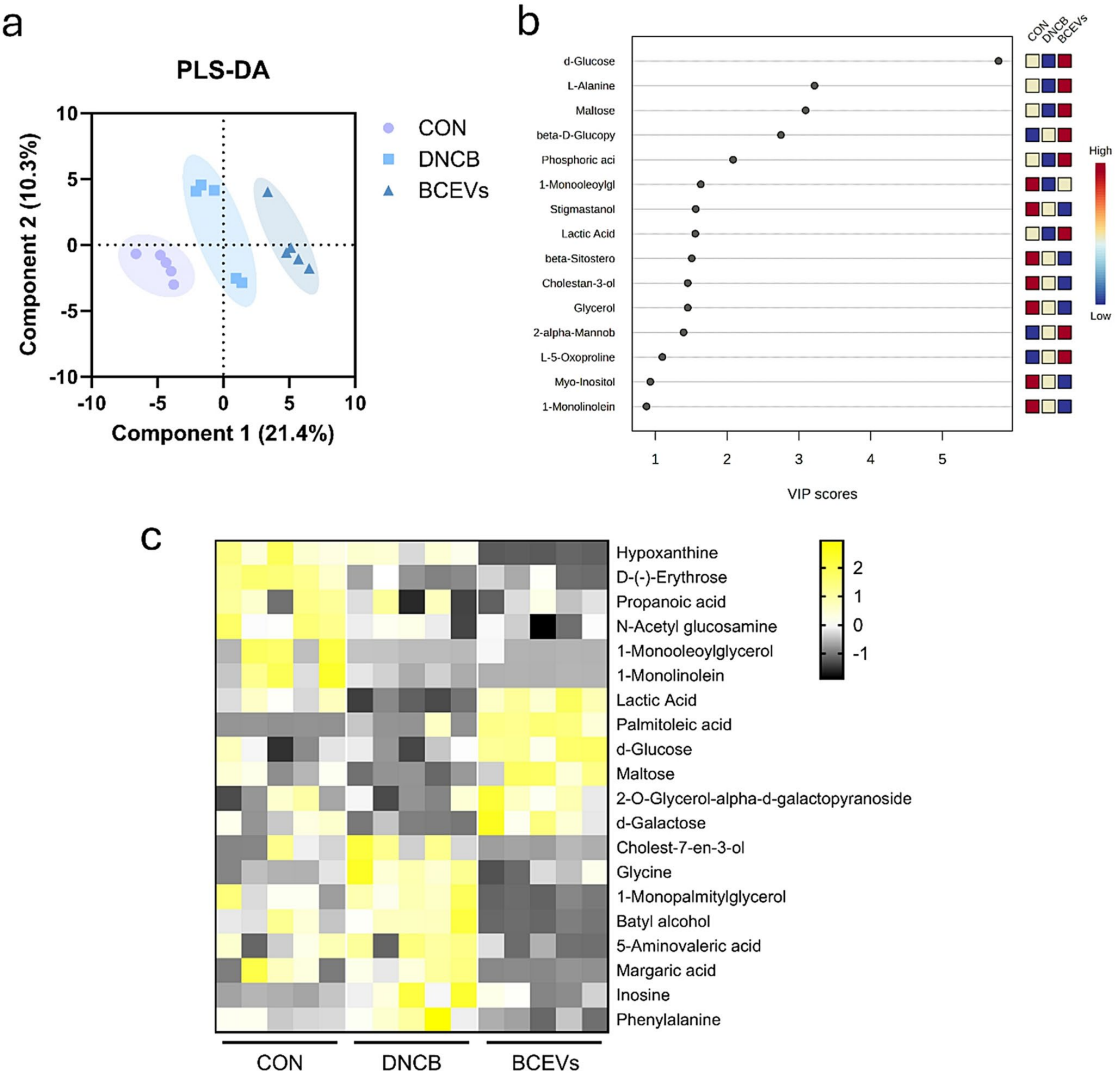
Metabolomic profiling reveals group differences in AD-like mouse model treated with BCEVs. (**a**) PLS-DA analysis of metabolites. (**b**) VIP score of PLS-DA analysis. (**c**) Heatmap of differences in metabolites among groups. AD, atopic dermatitis; CON, saline control; DNCB, AD control; BCEVs, AD inducing with BCEVs; PLS-DA, partial least squares discriminant analysis; VIP, variable importance in projection

**Fig. 7:**
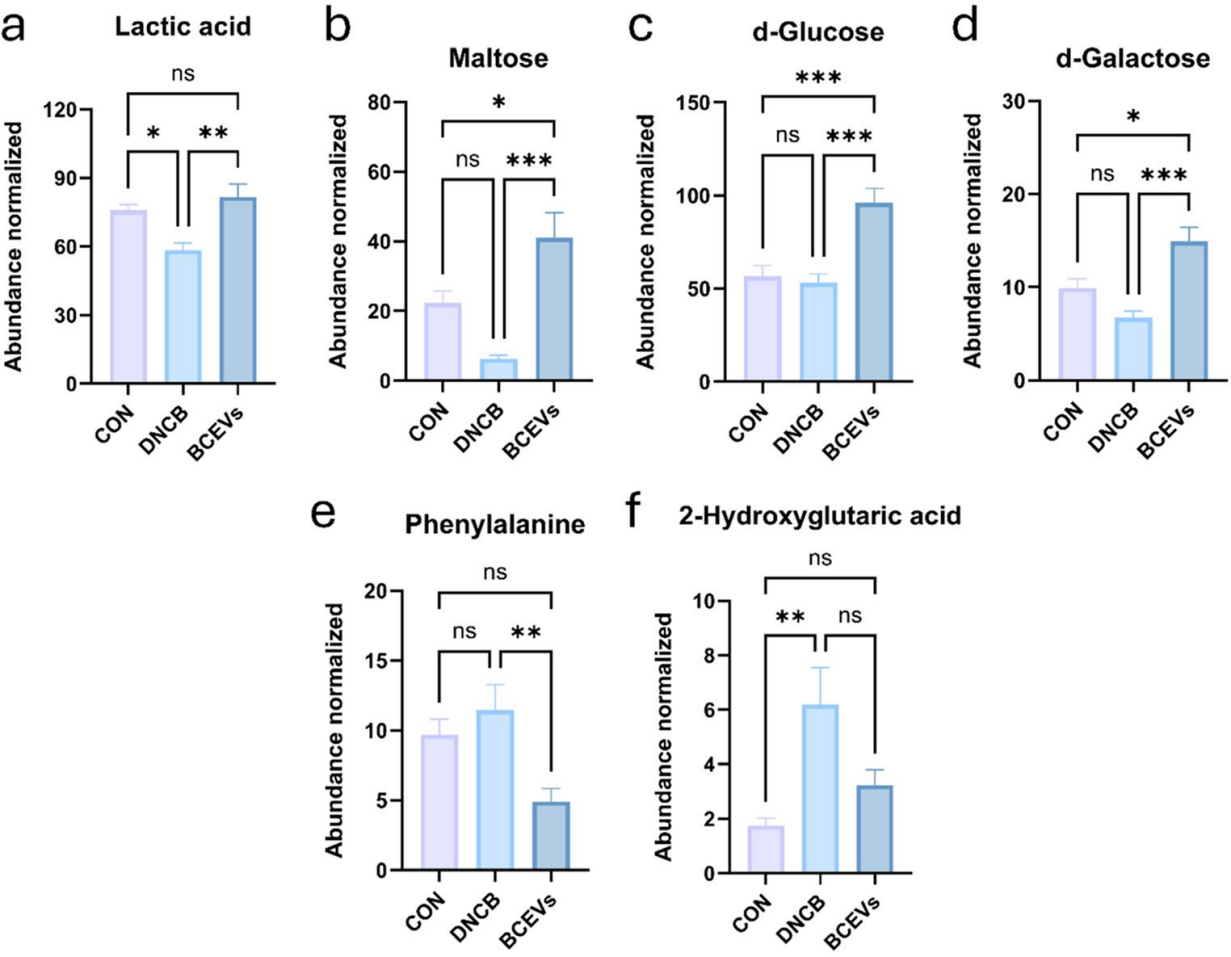
Comparison of significantly different metabolites. (**a**)–(**f**) Normalized abundance of lactic acid (**a**), maltose (**b**), d-glucose (**c**), d-galactose (**d**), phenylalanine (**e**), and 2-hydroxyglutaric acid (**f**) with different detections among groups. All data represent the mean ± standard deviations (*n* = 5). One-way ANOVA and Tukey’s test analyzed differences among multiple groups. Asterisks represent statistical significance at *p* < 0.05 (*), *p* < 0.01 (**), *p* < 0.001 (***), and *p* < 0.0001 (****), while *ns* indicates not significant. AD, atopic dermatitis; CON, saline control; DNCB, AD control; BCEVs, AD inducing with BCEVs

To investigate how BCEV-induced shifts in the gut microbiota affected the gut metabolome, a cecal metabolomic analysis was performed. PLS-DA showed that the groups clustered differently (Fig. 6a). As shown in the VIP score, d-glucose, L-alanine, maltose, phosphoric acid, and lactic acid were the metabolites that best represented the differences between groups (Fig. 6b). In comparing metabolite expression between groups, cholest-7-en-3-ol, glycine, batyl alcohol, 2-hydroxyglutaric acid, and phenylalanine, which were increased by AD induction, were significantly decreased by BCEVs (Figs. 6c and 7). Conversely, lactic acid was significantly decreased in the DNCB group but increased by BCEV treatment (Fig. 7a). Maltose and d-glucose were also found to increase in the BCEV treatment group compared with the other groups (Fig. 7b–d). These results indicated that BCEV treatment not only regulated the abundance of the gut microbiota but also affected the expression of the gut metabolome.

## Discussion

In this study, a DNCB-induced AD-like mouse model was used to assess the potential of BCEVs to modulate the gut microbiota and alleviate AD-like symptoms. Results confirmed a well-established AD mimetic model, including weight loss, increased skin thickness, mast cell and allergic responses, and intensified immune responses in DNCB-treated mice. In addition, BCEV treatment alleviated skin lesions and significantly reduced mast cell numbers, allergic reactions, and immune responses. Metagenomic analysis revealed a decrease in the abundance of the beneficial microbes *Lactobacillus* and *Bifidobacterium* in the DNCB group, which was restored by treatment with BCEVs. Metabolomic analysis specifically showed that lactic acid, which was lowly regulated in the DNCB group, was significantly increased upon BCEV treatment, and metabolites involved in the disrupted amino acid metabolism were restored. These results suggest that BCEVs could improve AD-like symptoms by restoring AD-induced gut microbiota imbalance.

In the pathophysiology of AD, allergic reactions play an essential role, mediated by major immune components such as mast cells, histamine, IgE, and the high-affinity IgE receptor (FcεRI, encoded by the Fcer1g gene) [30, 31]. When IgE binds to FcεRI on the surface of mast cells, mast cells are activated and secrete inflammatory mediators such as histamine and cytokines that enhance skin inflammation and itching [1, 32, 33]. In addition, TNF-α and the Th2 cytokines IL-4, IL-5, and IL-13 are secreted during mast cell activation upon stimulation by FcεRI [3, 34]. Previous studies have shown that treatment with *Pediococcus acidilactici*, a type of probiotic, reduces the number of mast cells and levels of serum IgE and attenuates the inflammatory response [35]. The oral administration of *Lactobacillus plantarum* reduced skin lesions, serum IgE levels, and Th2 cytokine production [28]. Furthermore, *L. plantarum* lysates ameliorate AD by inhibiting the production of IL-4 and IgE [36]. In addition, it has been reported that oral administration of *Limosilactobacillus fermentum* SLAM216 or its EVs alleviated AD-like symptoms and improved gut dysbiosis in AD-induced mice [26]. Similarly, *Lactobacillus reuteri* Fn041 has been reported to prevent AD in mice by inducing regulatory T cells and modulating the gut microbiota, particularly promoting *Lactobacillus* and *Akkermansia* [37]. In this study, BCEV treatment increased the abundance of beneficial bacteria such as *Lactobacillus* and *Bifidobacterium* among the gut microbiota, suggesting that these changes in the gut microbiota contributed to the decrease in mast cell activation and inflammatory response.

The aggravation of the AD symptoms is strongly linked to an imbalance in the gut microbiome. A previous clinical study reported an increase in the abundance of Firmicutes in the allergic disease group compared with the healthy group [38–40]. This study demonstrated that the AD-induced disruption to the Firmicutes/Bacteroidota ratio was restored by BCEV treatment. In addition, the gut microbiota imbalance characteristic of AD is accompanied by a decrease in diversity or *Lactobacillus* and *Bifidobacterium*, which are known to be beneficial bacteria. Previous studies have shown that the abundance of *Lactobacillus* is reduced in the gut of a mouse model of DNCB-induced AD [41, 42]. In addition, the abundance of the *Lactobacillus* group and *Bifidobacterium* tended to be lower in the gut microbiota of children with atopy, and the abundance of *Faecalibacterium prausnitzii* and *Akkermansia muciniphila* were significantly lower [43]. Notably, milk-derived EVs have been reported to modulate gut microbiota composition, particularly increasing the abundance of *Lactobacillus* and *Akkermansia* [13, 14, 44]. The potential to alleviate AD symptoms by regulating beneficial gut microbes highlights the need for further investigations into targeted microbial interventions [45, 46].

One of the mechanisms by which probiotic treatment alleviates AD is by increasing the levels of Th1 cytokines and inhibiting the production of IL-4 and IL-5 [47]. It is also associated with the differentiation of regulatory T cells in mesenteric lymph nodes [45]. Previous studies have reported that milk-derived EVs increase the number of Treg cells in the gut of DSS-induced colitis mice [13], and breast milk–derived miRNAs reportedly regulate the expression of Treg cells [48, 49]. Moreover, recent studies have demonstrated that milk-derived EVs or probiotics ameliorate inflammatory diseases, including AD, by regulating gut microbiota composition and suppressing Th2-mediated immune responses [13, 37, 50]. Consistent with previous findings, this study demonstrated that BCEV treatment ameliorated AD symptoms, which was attributed to a restored balance in the gut microbiota, evidenced by an increased abundance of *Lactobacillus*, and enhanced immune responses through the modulation of Th2 cytokines.

Recent research has highlighted the significant role of the gut microbiome and its metabolites in modulating immune responses and maintaining skin health [51]. Previous studies have shown that d-galactose ameliorates AD by modulating the gut microbiota [52]. Notably, certain gut-derived metabolites, such as indole derivatives from tryptophan metabolism, activate the aryl hydrocarbon receptor, leading to the attenuation of AD symptoms [53–55]. Herein, metabolomic analysis demonstrated that treatment with BCEVs ameliorated the imbalance in lactic acid and amino acid metabolism observed in the gut of the AD-induced model. Furthermore, BCEV treatment elevated the glucose, maltose, and galactose levels in the gut. This improvement is likely attributable to the increased abundance of intestinal lactic acid bacteria and *Bifidobacterium* following BCEV administration. Although the gut microbiota and its metabolites play a critical role in the pathogenesis and potential treatment of AD, further research is required to elucidate the specific impacts of the changes in particular gut metabolites on AD symptoms.

This study has several limitations that should be addressed in future research. First, as this study primarily focused on evaluating the in vivo functional effects of BCEVs, cargo profiling was not included. Further multi-omics approaches are needed to identify the specific bioactive components of BCEVs. Second, to validate the unique effects of BCEVs, future studies should include a whole colostrum-treated group for comparison. Third, only male mice were used to minimize biological variability; however, sex-specific effects should be evaluated in future studies. Finally, the experiments were conducted without positive and negative control groups, such as dexamethasone treatment or inactivated BCEVs. Although BCEV treatment significantly improved clinical symptoms, histological features, cytokine expression, and gut microbial composition, further studies including these controls will be necessary to more clearly validate the specific effects of BCEVs.

## Conclusions

**Fig. 8:**
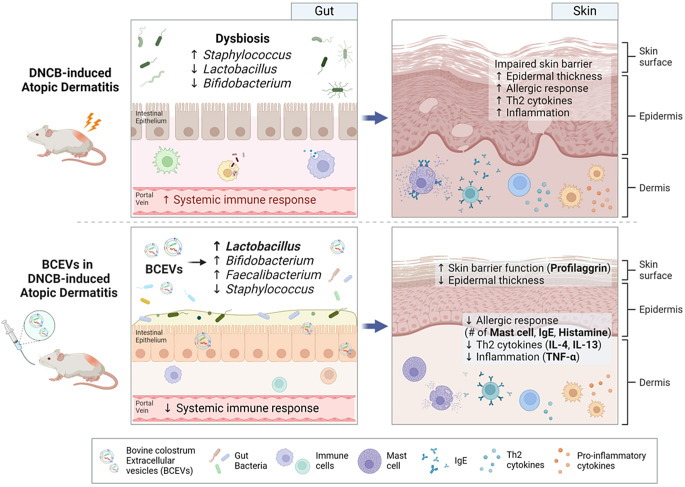
Schematic presentation of the functionality of BCEVs in atopic dermatitis. Bovine colostrum EVs attenuated systemic immune responses and skin allergic and immune responses and restored the skin barrier through the modulation of the gut microbiota, particularly *Lactobacillus* spp. DNCB, 2,4-dinitrochlorobenzene; Th2 cytokine, T helper type 2 cytokine; IgE, immunoglobulin E; Il, interleukin; Tnf, tumor necrosis factor

This study demonstrated that the oral administration of BCEVs effectively ameliorates DNCB-induced AD (Fig. 8). BCEV treatment alleviated skin lesions and mitigated the dysregulated immune response associated with AD. These therapeutic effects can be attributed to the restoration of intestinal bacterial balance, an increase in the abundance of beneficial bacteria such as *Lactobacillus*, and the regulation of the intestinal metabolome. These findings highlight the potential of BCEVs as a novel therapeutic approach for skin-related immune disorders by modulating the gut–skin axis. However, as this study was conducted in a controlled experimental model, further research is warranted to validate these effects in clinical settings and to elucidate the precise molecular mechanisms underlying the therapeutic impact of BCEVs. Future investigations should also explore the long-term safety and efficacy of BCEVs, as well as their potential applications in other immune-mediated conditions.

## Data Availability

The data that support the findings of this study are available from the corresponding author upon reasonable request.
